# Efficacy of ozonized water for the treatment of erosive oral lichen planus: a randomized controlled study

**DOI:** 10.4317/medoral.23693

**Published:** 2020-07-19

**Authors:** Federica Veneri, Elena Bardellini, Francesca Amadori, Giulio Conti, Alessandra Majorana

**Affiliations:** 1 Department of Oral Medicine, Dental Clinic, University of Brescia, Italy; 2 Department of Oral Surgery, University Vita-Salute San Raffaele, Milan, Italy

## Abstract

**Background:**

Management of erosive Oral Lichen Planus (eOLP) is challenging. Currently, topical corticosteroids are widely used as first-line therapy, but they might be associated with side-effects and incomplete clinical response. Among non-pharmacological strategies, ozone at low medical concentration has proven to induce a mild activation of protective anti-oxidant pathways, thus exerting therapeutic effects in many inflammatory diseases. The aim of this randomized controlled study was to investigate the effectiveness of ozonized water in association with conventional topical corticosteroids for the treatment of eOLP.

**Material and Methods:**

Fifty-one patients were included in the study and randomized into 2 groups: study group (n=26) included patients receiving ozonized water treatment; control group (n=25) included patients receiving placebo treatment (i.e. double-distilled water). Treatment protocol consisted of 1-minute oral rinses, repeated for 4 times, twice a week for 4 weeks. All patients received conventional corticosteroid topical therapy (betamethasone soluble tablets, 2 rinses/day for 4 weeks). Assessment of size of lesions, sign and pain scores was performed before treatment, after 2 weeks of treatment (T1) and at the end of 4-week treatment (T2). Efficacy Index (EI) of treatment, candidiasis and relapse rates were also recorded.

**Results:**

All patients experienced significant improvement of sign and pain scores with a higher rate of improvement in ozone-treated group (T1 improvement rates: Thongprasom 92.2% vs 28%; VAS pain 76.9% vs 32%; *p*<0.05). Pain and size reduction were significantly higher in ozone-treated group both at T1 and T2 (*p*<0.05). Ozone-treated group showed a higher EI at every time point (T0-T2: 72.77% vs 37.66%, *p*<0.01). Candidiasis (32% vs 11.5%) and relapse (40% vs 34.6%) rates were higher in control group, however the differences were not statistically significant.

**Conclusions:**

Within the limitations of this study, ozonized water seems to be effective as an adjunct therapy, in combination with topical corticosteroids, for the treatment of eOLP.

** Key words:**Oral Lichen Planus, OLP, ozone, treatment.

## Introduction

Oral Lichen Planus (OLP) is a chronic inflammatory disorder of the oral mucosa of unknown etiology, affecting approximately 2% of the population (1). It is considered a middle age disease (30-60 years of age) with a female to male ratio being 2:1 (2). The pathogenic mechanism consists of apoptosis of basal keratinocytes induced by CD8+ T cells because of an underlying immune disorder. Typically, the disease presents with multiple lesions, mostly with bilateral and symmetric distribution. Andreasen’s classification distinguishes 6 clinical presentations of OLP including reticular, plaque-like, atrophic (erythematous), erosive-ulcerous and bullous-erosive (3). Reticular, papular and plaque-like forms are the most common and usually painless; they are similar to other white disorders such as leukoplakia, appearing as white hyperkeratotic striae or plaques (4). Erosive and atrophic forms, on the contrary, are often associated with discomfort, pain and intolerance to spicy and hot food assumption. In addition, erosive long-lasting OLP is associated with a significant potential for malignant transformation with an estimated risk of 0.5-2% (2). Therefore, treatment of these forms and long-term monitoring are essential. Clinical management of erosive OLP (eOLP) is challenging, with no definitive cure available. Treatment aims primarily at abolishing the symptoms and at extending the duration of remission periods, but complete eradication of the disease is currently not achievable (5). A variety of treatments have been suggested, but there is no strong evidence for the effectiveness of any of them. Treatments include topical and systemic corticosteroids, topical calcineurin inhibitors, retinoids, immunosuppressants and anti-inflammatory coating gels (5). Currently, topical and systemic corticosteroids are widely used as first-line therapy, but they might be associated with side-effects, incomplete clinical response and frequent relapses (6). In particular, topical corticosteroids are considered the gold standard therapy for OLP, whereas systemic therapy is reserved only for multi-organ involvement or for severe cases resistant to conventional treatment (7). Nevertheless, long-term treatment with topical corticosteroids may be associated to adverse effects such as dysgeusia, tachyphylaxis, oral mucosa thinning, systemic absorption and secondary candidiasis (7,8).

The evaluation of other therapeutic strategies and non-pharmacological approaches is therefore essential. Photobiomodulation therapy (PBMT), previously known as low-level laser therapy (LLLT), has been widely used as a non-pharmacological alternative to corticosteroid therapy and it has proven effective, without any remarkable adverse effect (9,10). However, further studies with strict inclusion criteria, randomization, larger samples and precise standardization of the laser type and setting are required in order to evaluate long-term safety and efficacy (11). Among non-pharmacological strategies, the use of ozone (O3) as a complementary medical approach has progressively been increasing. Ozone is a highly unsTable atmospheric gas that rapidly decays into normal oxygen (O2). Although not being a radical molecule, O3 is a very strong oxidant and, due to this highly toxic property, it has been widely used as a disinfectant and germicidal agent, also for medical purposes (12). In addition, O3 administration as O2-O3 gas mixture has proven to improve metabolic activity and to exert therapeutic effects in numerous diseases (13).

Many T-cell mediated inflammatory diseases are driven by underlying imbalances in antioxidant response (14,15). Ozone at low medical concentrations induces a mild activation of protective anti-oxidant pathways, such as nuclear factor erythroid – related 2 (Nrf2) pathway, that help restore a redox homeostasis. (15) Nrf2 is able to modulate inflammation through the down-regulation of pro-inflammatory cytokines synthesis, reactive oxygen species (ROS) levels and transcriptional activity of nuclear factor kappa-B (NF-kB). The modulation of ROS levels by Nrf2 pathway is crucial in maintaining a proper T-cell mediated immunity (15,16). As a result, increased Nrf2 expression limits T cell activation and controls the differentiation of inflammatory T cell subsets, skewing the immune response towards more anti-inflammatory phenotypes (14,17). Unfortunately, the application of ozone therapy is still limited due to the numerous doubts about its possible toxicity (18). However, recent studies have helped to clarify that exposure to low O3 concentrations stimulates cell protective pathways and nuclear transcription without inducing cell damage and altering cell proliferation and survival (18,19). Given its therapeutic mechanisms, we hypothesize that ozone therapy can decrease the severity of eOLP symptoms, manage the risk of candidiasis superinfection and induce a faster healing of the lesions. Ozonized water was the chosen formulation as it overcomes the issues of gaseous ozone, including gas dispersion, potentially associated with risk of inhalation toxicity and reduction of local effectiveness.

The aim of this study was to investigate the use of ozonized water for the treatment of eOLP, in association with conventional topical corticosteroids application, in terms of efficacy index, clinical scores (i.e. VAS pain, size of lesions and Thongprasom scores) and candidiasis infection and relapse rates. To the best of our knowledge there are no similar studies.

Material and methods

- Sample selection

This study was carried out at the Dental Clinic of the University of Brescia, Brescia - Italy January 2018 to December 2019, including a 3-month follow-up. Fifty-five consecutive patients with eOLP were enrolled.

Inclusion criteria were: (a) histopathological diagnosis of OLP, according to the conventional WHO criteria (b) clinical erosive form, according to the clinical criteria of van der Meij and van der Waal (c) symptomatic lesions (20). Exclusion criteria were: (a) lesions showing OLP and dysplasia (b) lesions showing OLP and candidiasis (c) oral lichenoid lesions (d) patients who underwent corticosteroids or other immunosuppressive treatment. Data concerning age, sex and general medical history were collected.

- Study design

This study was designed as a randomized placebo-controlled study. Patients were randomized by computer code into two groups. Group A included patients receiving ozonized water treatment (Aquolab Professional, Sweden & Martina S.p.A, Padova, Italy) i.e. 1-minute oral rinses (double-distilled water to ozone ratio being 2:3), repeated for 4 times, twice a week for 4 consecutive weeks, for a total of 8 applications; group B included patients receiving placebo treatment, i.e. of double-distilled water oral rinses, with the same duration and timing. Water ozonization was carried out at each appointment, 5 minutes before application.

All patients received conventional corticosteroid topical therapy (betamethasone sodium phosphate 500 mg soluble Tablets, 2 rinses par day for 4 weeks).

Patients were evaluated before the treatment (T0), after 2 weeks (T1), at the end of the treatment (T2) and after 3 months as a follow-up (T3).

OLP clinical course was assessed by measuring severity of pain, lesions size, clinical signs and efficacy of the treatment. Clinicians who evaluated the outcomes were blinded to the allocation group.

- Pain scoring

The severity of pain was determined using a visual analogue scale (VAS) from 0 to 10 where 0 corresponds to “no pain” and 10 to “the worst possible pain”. The symptoms data were then scored according to the following classification: score 3: severe pain/discomfort (7<VAS<10); score 2: moderate pain/discomfort (3.5<VAS<7); score 1: mild pain/discomfort (0<VAS<3.5); score 0: without pain/discomfort (VAS=0) (21).

- Clinical signs and size of lesions

The lesion size was defined as the main diameter (mm) of the worst atrophic and erosive lesion of OLP, measured with a periodontal probe. The scoring was performed by two calibrated clinicians.

The change in clinical signs was assessed through Thongprasom sign scoring system as follows: score 5: white striae with erosive area >1 cm2; score 4: white striae with erosive area <1 cm2; score 3: white striae with atrophic area >1 cm2; score 2: white striae with atrophic area <1 cm2; score 1: white striae only; score 0: no lesions, normal mucosa (22).

- Efficacy of the treatment

Treatment efficacy index (EI) was calculated, using the following formula:

[(Total score of the lesion before treatment – Total score of the lesion after treatment) / Total score of the lesion before treatment] X100

The EI was categorized into 5 rank scale as follows: healed: 4: EI=100%; marked improvement: 3: 75%≤EI<100%; moderate improvement: 2: 25%≤EI<75%; mild improvement: 1: 0<EI<25%; no improvement: 0: EI=0 (5).

VAS and Thongprasom improvement were defined as transition to a lower VAS score and Thongprasom score class, respectively.

Possible candidiasis infection, diagnosed upon clinical signs and symptoms along with the presence of candida organisms by exfoliative cytology, was also recorded during treatment.

Relapse rate was also recorded 3 months after the end of the treatment (T3) and it was defined as worsening of at least one between VAS score or Thongprasom signs score.

- Statistical analysis

All data were recorded in Microsoft Excel datasheets and statistical analysis was performed using IBM SPSS Statistics (v25, Inc, Chicago, IL, USA). Descriptive analysis was carried out and data were presented as frequency, percentage, mean, standard deviation, median, interquartile range. The Kolmogorov-Smirnov test was used to assess data distribution. As data did not exhibit normal distribution, non-parametric tests were chosen. U-Mann-Whitney test was used to evaluate any difference in pain and clinical scores between groups and Wilcoxon matched paired test was used to determine any difference at different time points. Percentages of EI values, improvement, relapse and candidiasis rates were compared using Chi-square or Fisher’s exact test. Estimating a moderate improving (E.I. 2) at the end of the treatment in 90% of the cases for the ozone-treated group and in 55% for the control group, the minimum number of patients for the study, assuming alpha 0.05 and beta 0.20 (study power = 80 %), was calculated to be 50 (at least 25 per group) (9).

## Results

Fifty-five patients affected by atrophic-erosive OLP were recruited for the study according to inclusion criteria. Four patients were lost to T2 scheduled appointments and follow-up and were excluded from the study. Therefore, a total of 51 patients (35 females and 16 males) were included. The mean age of the patients was 65.14 (range 46-83). The oral sites involved were buccal mucosa, gums and dorsum of the tongue. Initial demographic and clinical features were similar for both groups (*p*>0.05) (Table 1).

**Table 1:**
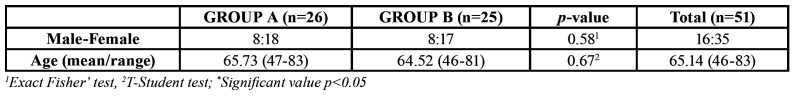
Demographic characteristics of the patients.

Reduction in signs and pain scores at different time points throughout the study, within the same group, was statistically significant. (Wilcoxon paired test, *p*<0.001).

**Figure 1:**
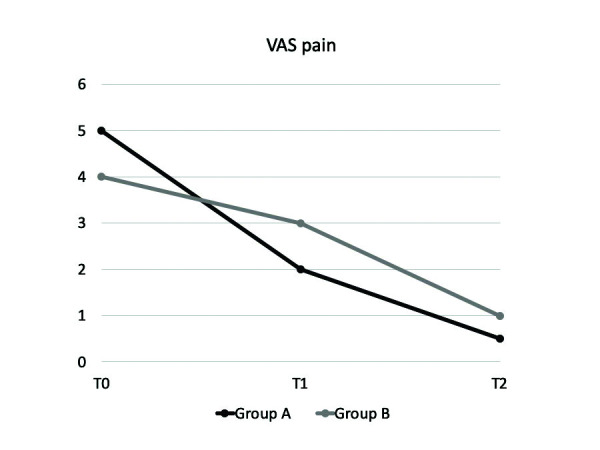
VAS pain median values over time.

With regards to pain evaluation, VAS clinical features are displayed in Table 2. Pain reduction was significantly higher in group A both at T1 and T2 (*p*<0.05). (Fig. 1) Most of the patients reported a VAS score of 2 (moderate pain) at T0, which lowered to score 1 or 0 at T2. VAS score difference between groups was statistically significant both at T1 and T2 (*p*<0.05).

All patients experienced a significant improvement of symptoms throughout the treatment, however, both at T1 and T2 a higher rate of improvement was found in group A, with a statistically significant difference at T1 (*p*=0.001).

**Figure 2:**
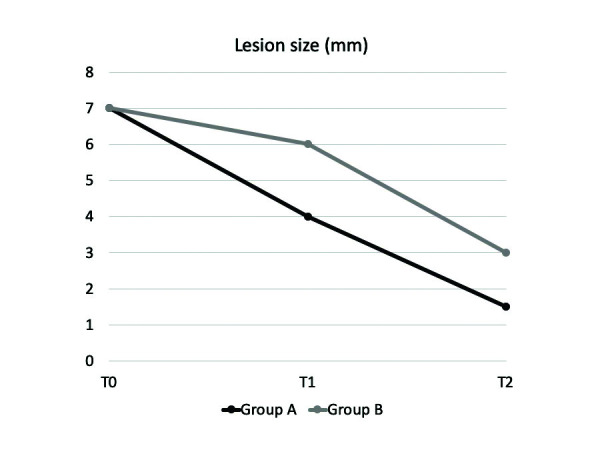
Size of lesions (median, mm) over time.

**Figure 3:**
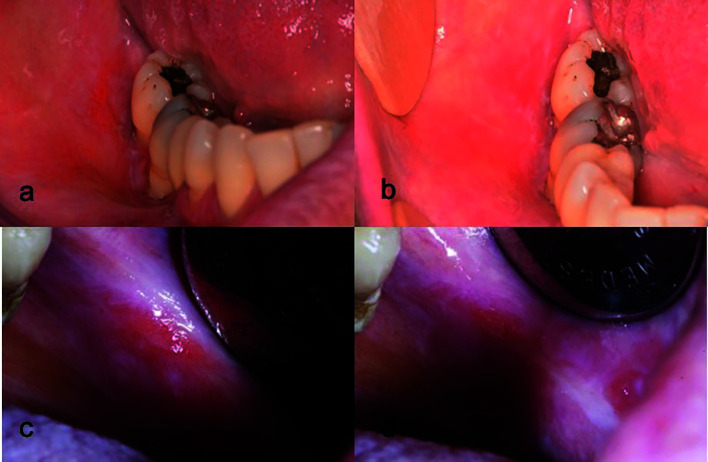
Clinical presentation of emblematic cases from study group (a; b) and from control group (c; d), before (a; c) and after treatment (b; d) respectively. (Study group: betamethasone rinses associated with ozonized water rinses. Control group: betamethasone rinses and placebo (double-distilled water) rinses).

Size of the lesions ranged from 2 to 20 mm. Size features and Thongprasom signs scores are displayed in Table 3. The difference in reduction of lesions size between groups was statistically significant at T1 (*p*<0.05) and T2 (*p*=0.001). (Fig. 2) Most patients showed Thongprasom score 2 and 4 at T0, which averagely improved to grade 1 after treatment (T2). Thongprasom signs score improvement rate was higher in group A, but a statistically significant difference was found only at T1 (*p*<0.001) (Fig. 3).

**Table 2:**
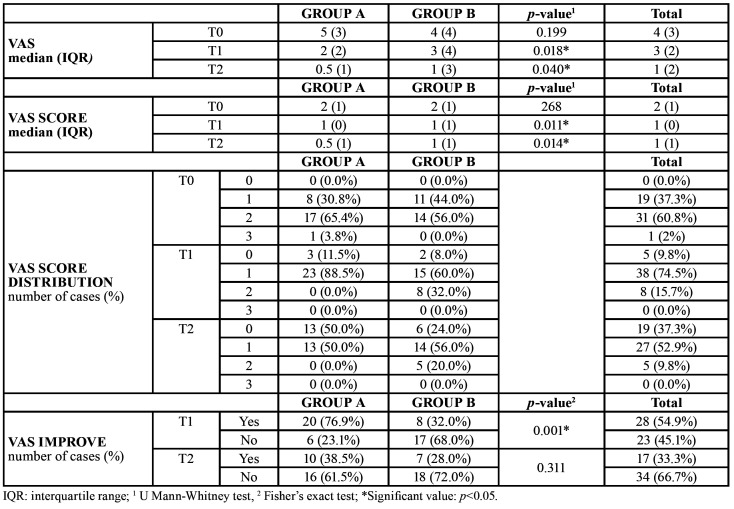
VAS pain distribution at T0, T1, T2.

**Table 3:**
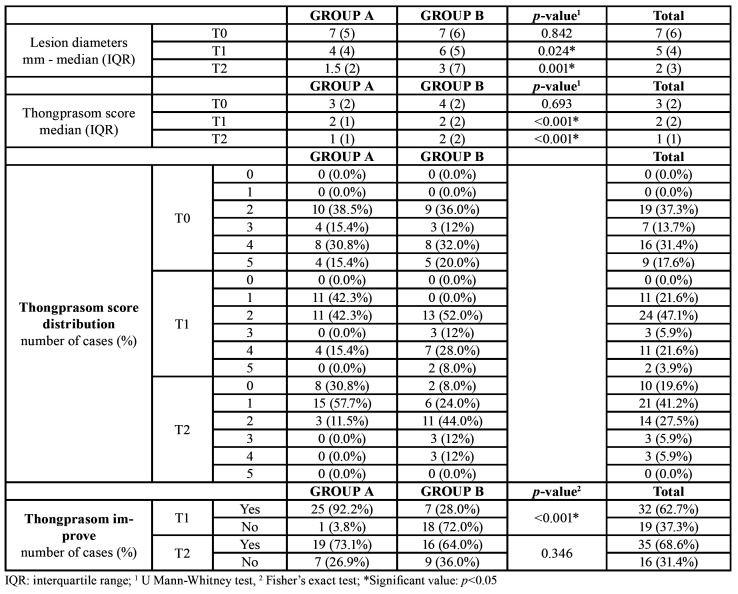
Lesion size and signs score (Thongprasom score) at T0, T1, T2.

Efficacy Index (EI) data, relapse and candidiasis rates are shown in Table 4. Most of the patients showed a moderate improvement (EI=2) at the end of the treatment in both groups. EI of the treatment was significantly higher for group A (*p*<0.05) at every time point.

Relapse rate at T3 was higher in group B (40%) compared to group A (34.6%), however the difference was not statistically significant (*p*=0.457).

The rate of candidiasis infection during treatment was not significantly different (*p*=0.075) between groups, however a higher number of patients was affected in group B (n=8) if compared to group A (n=3).

**Table 4:**
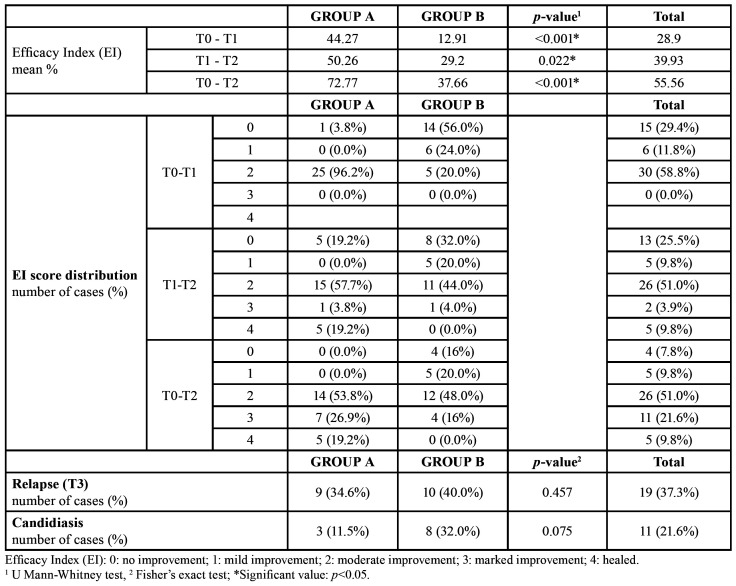
Efficacy Index (EI), relapse rate and candidiasis rate distribution.

## Discussion

OLP is a common chronic immunological disease and its management is still challenging for clinicians. Currently there are no resolutive treatments available and the main aim of therapy is to relieve inflammation, pain and discomfort associated with the erosive forms of the disease. In addition, long lasting atrophic-erosive OLP are associated with a significantly higher potential for malignant transformation than reticular-keratotic forms (2). Topical corticosteroids are widely accepted as first line-therapy for symptomatic OLP. However, prolonged use of this group of medication should be avoided, as it is associated with many adverse effects such as dysgeusia, systemic absorption, secondary fungal infections and increased risk of malignancies because of immune system suppression (8). The introduction of alternative safe treatment methods is, therefore, strongly required. PBMT has long been used for many oral inflammatory diseases and its efficacy is widely supported by clinical practice. PBMT seems to induce acceleration of wound healing, anti-inflammatory effects, stimulation of cellular metabolism, immune-modulation, vasodilatation and analgesic effects (23). However, the efficacy of PBMT depends on many parameters such as power, wavelength, intensity, exposure time, modality of application and treatment protocol. Therefore, PBMT efficacy is still controversial and further studies are needed (5,11).

Among non-pharmacological strategies, ozone use has been increasing as a complementary medical approach. Ozone has many therapeutic properties, including immune-modulation, pain relief, promotion of biosynthetic activities, antioxidant, antimicrobial and wound healing properties. In addition, it enhances microcirculation in tissues (13). Kazancioglu *et al*. found that, although both ozone and laser therapies had a positive effect on bone formation in rat calvarial defect, ozone therapy was more effective than PBMT (24). A study by Erdemici *et al*. demonstrated that ozone has beneficial effects on wound healing both on hard and soft tissue in an experimental model, as it reduced inflammation and edema by activating biochemical mechanisms and antioxidant systems (25). In the present study we obtained promising results in terms of reduction of signs and symptoms associated to atrophic-erosive OLP. Lesion size, Thongprasom score and pain significantly decreased in the group treated with ozone and topical corticosteroids in comparison to the group treated with corticosteroids alone. Our results are consistent with Mostafa *et al*. who found that gaseous ozone treatment in combination with topical corticosteroids was more effective than corticosteroids alone in reducing sign score and pain of OLP throughout the treatment (26). Bayer *et al*. found that both PBMT and gaseous ozone treatment have positive effects in the treatment of chemo-radiotherapy induced oral mucositis, being PBMT more effective than ozone. These results may be related to the duration and dose of the laser and ozone applications. Different laser wavelengths and different duration, dose and modality of application of ozone may change the results (23). Unlike the aforementioned studies, we chose ozonized water over gaseous ozone. Gaseous ozone requires a direct careful and controlled application. In fact, possible gas dispersion might be associated to toxicity risk by inhalation in addition to reduction of local effectiveness. Moreover, the mechanism used for gaseous ozone production (corona discharge) is associated to relative or absolute contraindications (e.g. pregnancy, pacemaker, neurological diseases) that must be taken into account. Ozonized water rinses might overcome these issues, since ozone is produced out of the patient’s mouth and immediately conveyed into the medium. Half-life of ozone in double-distilled water is approximately 10 hours. In our protocol, ozonized rinses were performed 5 to 10 minutes from production in order to maintain the original concentration as much as clinically achievable. This formulation allows to reach lesions located up to the oropharynx and might be a rapid, safe and easy-to-use alternative to gaseous ozone and most patients showed a high grade of acceptance of the treatment.

The reason for the effectiveness of ozone therapy in OLP could be found in the ethiopathogenesis of this disease, mediated by CD8+ T lymphocytes. The basal layer disruption depends on the cytotoxic effects of the T cells in variable distribution at the sub-epithelial inflammatory infiltrate. A pivotal role in the pathogenesis of this long-lasting inflammatory process is played by the activation of nuclear factor kappa B (NF-κB), a primary transcription factor which, upon translocation to the nucleus, binds to promoter regions of different genes encoding immune and proinflammatory mediators (16). Ozone therapy induces the modulation of ROS levels and transcriptional activity of NF- κB by Nrf2 pathway and limits T cell activation, inducing more anti-inflammatory phenotypes (14,17). The effect of the ozone therapy on the activity of CD8+ T cells, that trigger apoptosis of oral epithelial cells in OLP, may explain the reduction of the damage on the basal keratinocytes.

Relapse rate resulted lower in the ozone-treated group. Though not statistically significant, This outcome might suggest that absence of inflammation is maintained further in time, since ozone improves the oxygen conveying limit of blood, causing better metabolism of cells and tissues and positively affects immune response In fact, as Noel at al. previously demonstrated on mouse models, T cell–specific activation of Nrf2-regulated antioxidant response appears to help in the maintenance of a low proinflammatory environment and optimal T cell function that subsequently results in reduced oxidative and inflammatory tissue injury (27). A limited 3-month follow-up was chosen for the purpose of this study, as it is considered to be reasonable both to intercept the most recurrent forms of OLP and to limit the other confounding factors that may be associated with long-term follow-up (28). However, such a short follow-up might not fully describe OLP clinical course. Being OLP a chronic condition that will most likely recur over time, the data reported in this study might underestimate the real relapse rate. Therefore, well-designed studies with longer follow-ups and experimental models investigating long-term effects of ozone application are recommended.

Being OLP a disease at risk of malignant transformation, a consideration about the safety of the ozone therapy is due. Scully *et al*. identified the basis of the malignant transformation of OLP in the accumulation in the oral epithelium of the inducible nitric oxide synthase with 8-oxodG (8-nitroguanine and 8-oxo- 7,8- dihydro-2¢- deoxyguanosine), which could reflect the oxidative and nitrative DNA damage (29). While strong oxidative stress fails to properly activate anti-oxidant intracellular pathways, mild oxidative stress induces a controlled increased expression of anti-oxidant Nrf2. The direct and indirect molecular targets of Nrf2 delineate a complex network of biological processes that reduce aberrant inflammation, preserve cell homeostasis and promote cell reparative programs, thus protecting cells from DNA damage, preventing the primary trigger of neoplastic transformation and, therefore, supporting its safety for the treatment of potentially malignant conditions (14).

Ozone also shows marked antimicrobial activity. In fact, in the current study candidiasis rate resulted lower in ozone-treated group. This was in line with a study conducted by Arita *et al*. who concluded that the use of ozonized water might be useful in reducing the counts of oral *Candida* Albicans on resin denture plates due to its strong antifungal properties (30).

Within the limitations of this study, it can be concluded that ozonized water rinses can be combined with topical corticosteroids as an adjunct therapy, resulting safe and effective in the treatment of symptomatic eOLP. However, additional studies are recommended, taking into account wider samples and long-term follow-ups, and, possibly, comparing ozonized water alone versus conventional topical corticosteroid treatment. Being ozone application relatively new in oral medicine, further studies might also be useful to identify the most appropriate treatment protocols for inflammatory oral diseases.
